# The Food4toddlers study - study protocol for a web-based intervention to promote healthy diets for toddlers: a randomized controlled trial

**DOI:** 10.1186/s12889-019-6915-x

**Published:** 2019-05-14

**Authors:** Margrethe Røed, Elisabet R. Hillesund, Frøydis N. Vik, Wendy Van Lippevelde, Nina Cecilie Øverby

**Affiliations:** 1 Department of Public Health, Sport and Nutrition, Faculty of Health and Sport Sciences, University of Agder, PO box 422, 4604 Kristiansand, Norway; 2 Department of Marketing, Innovation and Organization, Ghent University, Tweekerkenstraat 2, 9000 Ghent, Belgium

**Keywords:** Randomized controlled trial, Parental feeding practices, Food environment, Eating environment, Toddlers, eHealth, Shopping behavior

## Abstract

**Background:**

Eating habits are established during childhood and track into adolescence and later in life. Given that these habits have a large public health impact and influence the increasing rates of childhood obesity worldwide, there is a need for effective, evidence-based prevention trials promoting healthy eating habits in the first 2 years of life.

The aim of this study was to develop and evaluate the effect of an eHealth intervention called Food4toddlers, aiming to promote healthy dietary habits in toddlers by targeting parents’ awareness of their child’s food environment (i.e., how food is provided or presented) and eating environment (e.g., feeding practices and social interaction). This paper describes the rationale, development, and evaluation design of this project.

**Methods/design:**

We developed a 6-month eHealth intervention, with the extensive user involvement of health care nurses and parents of toddlers. This intervention is in line with the social cognitive theory, targeting the interwoven relationship between the person, behavior, and environment, with an emphasis on environmental factors. The intervention website includes recipes, information, activities, and collaboration opportunities. The Food4toddlers website can be used as a mobile application. To evaluate the intervention, a two-armed pre–post-follow-up randomized controlled trial is presently being conducted in Norway. Parents of toddlers (*n* = 404) were recruited via social media (Facebook) and 298 provided baseline data of their toddlers at age 12 months. After baseline measurements, participants were randomly allocated to an intervention group or control group. Primary outcomes are the child’s diet quality and food variety. All participants will be followed up at age 18 months, 2 years, and 4 years.

**Discussion:**

The results of this trial will provide evidence to increase knowledge about the effectiveness of an eHealth intervention targeting parents and their toddler’s dietary habits.

**Trial registration:**

ISRCTN92980420. Registered 13 September 2017. Retrospectively registered.

## Background

It is acknowledged that long-term health has an early developmental origin [1, 2]. The period from conception until 2 years of age, “the first 1000 days of life”, is recognized as a critical period for growth and development as the developing child adapts both metabolically and behaviorally to its nutritional and overall environment via gene expression and epigenetic mechanisms [3, 4]. Given that an unhealthy diet is one of the key risk factors for overweight, obesity, and other related noncommunicable diseases (NCDs) [5], diet quality during these formative years may strongly influence the child’s life-long health trajectory [6].

In Norway, as in other countries, unhealthy dietary patterns characterized by low intake of fruits and vegetables and high intake of non-core foods and beverages, are observed among toddlers [7–11]. In addition, at 12 months of age, about 80% of Norwegian children eat commercial baby food, with the main food intake for more than 15% of children aged 24 months still coming from jarred foods [12, 13]. Furthermore, studies have shown unhealthier dietary patterns in young children from families with lower socioeconomic status (SES) than those with higher SES [14–16]. There is a social gradient in child diet quality disfavoring the long-term health of children with lower SES [16, 17].

Parents are the gatekeepers of foods served during the first years of life and they have a unique role in shaping their child’s dietary behavior [18, 19]. Dietary preferences (likes and dislikes) and food habits established early on reflect to a large extent parental feeding practices, such as the type and variety of foods offered during the first 2 years of the child’s life [18]. Early dietary habits have been shown to track to later in childhood and adulthood [7, 20]. Fostering healthy dietary habits is therefore crucial to long-term health and obesity prevention [20]. Whether healthy or unhealthy dietary preferences are established depends on what, when, and how the child is fed [18]. To promote the internal regulation of energy balance, parents should be responsive to a child’s hunger and satiety cues during meals and feeding [21, 22]. One-year old children are capable of eating foods consumed by the whole family, and the development of self-feeding skills should be encouraged in this period [23].

Parental feeding practices are influenced by nutrition knowledge, family meal practices, and overall food preparation and parenting skills [24]. Non-responsive feeding (i.e., excessively controlled feeding, indulgent feeding, or uninvolved feeding) has been linked to childhood obesity [25]. Campbell and Crawford [26] identified several factors in the family environment to be important for children’s diet, including parental food preferences and beliefs, children’s food exposure, role modeling, media exposure, and child–parent interactions around food. In another study, those authors demonstrated several aspects of the family’s food environment (e.g., TV viewing and shared meals) to be associated with child dietary characteristics that are likely to promote fatness [27].

Lobstein et al. [28] claimed that the food environment is the leading factor driving obesogenic behaviors. The *food environment* refers to factors that directly relate to how food is provided or presented such as its salience, structure, packaging or portion size, and how it is served [29]. The food environment is further divided into *macro-scale* (e.g., food shopping outlets) and *micro-scale* (e.g., home environment). The *eating environment* refers to factors that are independent of foods, such as social interactions around meals, atmosphere, and the time of day that meals are eaten [29]. Roberto and Kawachi [30] found that many of people’s daily eating habits are guided by default options, e.g., large portion sizes in restaurants. According to Roberto and colleagues [31], current food environments exploit our biological, psychological, social, and economic vulnerabilities by making it easier to access and eat unhealthy non-core foods that either increase overall energy intake or replace healthy core foods in the diet. In-store environmental factors (e.g., the placement of healthy foods) influences parents’ choices when shopping [32]. The food industry produces jarred food, squeezable fruit pouches, and baby porridge for children up to the age of 24 months and older that are often packed in colorful, attractive wrappings and marketed as a healthy choice. These high-cost products are often strategically placed in the store. These foods are unnecessary for toddlers and do not meet the child’s need for different texture, flavors, and dietary variety [33]. Addressing awareness of how both the macro- and micro-scale food environments affect choices regarding foods and feeding is important, to help parents make more informed choices.

Although interventions at early ages are decidedly needed, they are scarce [34–37]. Two dietary intervention trials in Australia have addressed the parental role in shaping healthy eating environments for young children [22, 38]. The cluster-randomized INFANT study focused on parenting skills related to diet and physical activity in children aged 3–18 months, and resulted in lower consumption of sweet snacks and less daily television time [39]. The NOURISH trial, a community-based intervention targeting early parental feeding practices in 4- to 16-month old children [22], reported higher use of protective feeding practices conducive to the development of healthy eating patterns and healthy growth in the intervention group compared with the control group [40]. To our knowledge, no studies have applied eHealth approaches targeting diet in young age groups via the parents [34, 35, 41].

Interventions using smartphones and computers have a high potential to reach a large number of people, including those with low SES. Such interventions are cost-effective, flexible, have a low participant burden, and may be more visually appealing and engaging [42]. Therefore, we developed an eHealth intervention called Food4toddlers, with a mobile application (app) version for use with a smartphone.

## Objectives and outcomes

The aim of this study was to develop and evaluate the effect of an eHealth intervention called Food4toddlers, aiming to promote healthy dietary habits in toddlers by targeting parents’ awareness of their child’s food and eating environments.

### Primary outcomes

Primary outcomes of the study are child diet quality and food variety assessed at baseline and after the intervention.

### Secondary outcomes

Secondary outcomes include the food and eating environments conceptualized as: parental feeding practices, family meal settings (frequency of meals, meal distractions), food choice, awareness of the food environment (at home and in the grocery store), availability and accessibility of food at home, food preparation and planning, and child weight and length.

## Methods/design

### Study design

**Fig. 1:**
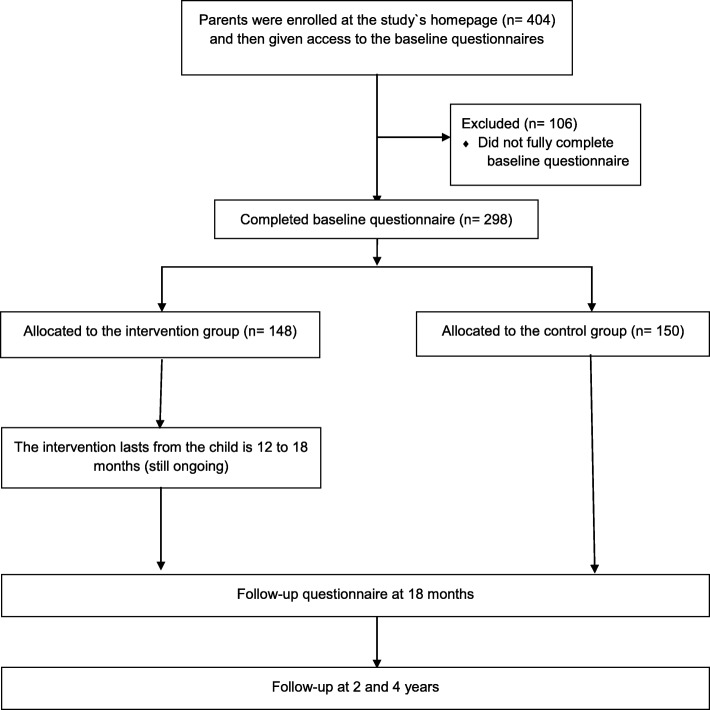
Flow chart of the Food4toddlers study design

This study is a randomized controlled trial to evaluate the effect of the Food4toddlers intervention, in which the intervention group has access to the Food4toddlers intervention website and the control group does not, see Fig. 1. Children in the intervention and control groups receive their usual care at community child health centers, which normally includes three consultations with a health care nurse for children between 12 and 18 months of age. The study started in August 2017 and is ongoing.

Participating parents complete questionnaires at baseline, post-intervention (end of intervention, after 6 months), and at two follow-ups (i.e., when their child turns 2 and 4 years old). The intervention runs in waves, and the first group started the intervention in September 2017. New groups were started every month through February 2018.

### Study sample and recruitment

The study population comprised children close to 12 months and one of their parents. To be included in the study, parents had to have a child born between August 2016 and April 2017 and the parents had to be literate in Norwegian. Participants were recruited via Facebook. A short video was launched on Facebook with a link to the project website containing information about the project and the opportunity to sign up. The recruitment period lasted 5.5 months from mid-August 2017 to January 2018. In total 404 parents were recruited. The month before the child reached age 12 months, the enrolled parent received an e-mail with a link to a questionnaire. Three reminders on e-mail were sent to non-responders the following weeks. We included a total of 298 parents who responded to more than half of the survey questions. After they had completed the baseline questionnaire, participants were randomized according to an SPSS-generated randomization list prepared of NCØ, of 500 to the intervention and control group (SPSS version 24.0). The first author was the one who enrolled participants and assigned participants to the intervention group and control group. Among the participants who answered baseline and post intervention questionnaires, ten participants were selected to receive a gift card of 1000 Norwegian kroners. Demographic characteristics of the sample are provided in Table 1.

**Table 1:** Characteristics of participating parents and children

		Intervention (*n* = 148)	Control (*n* = 150)
Parents (*N* = 298)	Mother/father/other (n)	144/4/0	148/0/2
	Age (year), mean (SD))	31.5 (4.4)^a^	31.9 (4.0)
	Height, mean kg (SD)	168.7 (6.0)	168.1 (5.9)^b^
	Weight, mean cm (SD)	70.8 (14.3)	71.1 (14.8)^b^
	BMI, mean (SD)	24.9 (4.6)	25.1 (4.8)^b^
	Two adult household (%)	98.0	96.7
	Family members (n), mean (SD)	3.60 (1.0)	3.65 (0.87)
	Born in Norway (%)	89.2	83.2^a^
Education		^a^	^a^
	Upper-level secondary school or less (%)	12.2	11.4
	College/university (≤4 years) (%)	31.3	36.9
	College/university (> 4 years) (%)	56.5	51.7
Geographic residence			
	Northern Norway (%)	4.5	6.7
	Central Norway (%)	10.8	10.7
	Western Norway (%)	23.0	20.7
	Southern Norway (%)	16.2	20.0
	Eastern Norway (including Oslo) (%)	44.6	42.0
Child			
	Age (months (SD))	10.9 (1.3)	10.8 (1.2)
	Girls (%)	46.6	43.3

^a^one missing, ^b^two missing

Sample size was calculated for one of the primary outcomes, child diet quality. As we have no data on healthy eating score for the Norwegian toddler population, we used the data of Angelopoulos and colleagues [43], which showed a mean healthy eating score of 60.5 among children (SD 9.0). We considered a 3-point difference in such a score between the intervention and control groups to be relevant from a public health perspective. We calculated that 142 children in each group would be required to demonstrate statistical significance with a statistical power of 80% and α of 5%. Assuming loss to follow-up of 40%, we aimed to recruit 237 parents in each group.

### Theoretical framework

This study was developed using the basic steps from the Model of Planned Promotion for Population Health, which recognizes the importance of evidence- and theory-based intervention planning [44]. The model builds on the Theory of Planned Behavior [45] and Social Cognitive Theory [46]. Health behavior theories have had a major focus on cognitive determinants, but newer models are addressing the relationship between behavior and environment [47, 48]. As suggested by Brug and colleges [44], a focus on how to promote action rather than mere motivation is emphasized. The present intervention is in line with the social cognitive theory, targeting the interwoven relationship between the person, the behavior, and the environment [49], with an emphasis on environmental factors. Research suggests that environments influence us at a basic level of which we are unaware and that we do not monitor [29, 50]. In this project, we aim to make parents aware of how the environment influences them and render them more conscious about the over 200 food choices they make on behalf of their children throughout their daily routines [29].

### Intervention development and user involvement

With the Food4toddlers intervention, we aim to influence child diet quality and food variety by targeting the main caregivers, the parents, and their awareness of the food and eating environments. The intervention outline was developed based on a literature review, extensive user involvement, and in-depth thematic discussions among the project group. Users in the development phase were parents of toddlers and health care nurses who were involved in several steps of the development of Food4toddlers. The first step in development was to contact public health nurses to get an overview of the questions that parents tend to ask about diet and nutrition, potential challenges, and how parents might perceive the potential need for online information. Three interviews were conducted (one face to face, two by telephone). We further conducted a focus group interview with health care nurses at their workplace, followed by an individual telephone interview to further elaborate on what health care nurses perceived as the most customary questions asked by parents regarding diet. One of the nurses worked in a disadvantaged community that included a large non-native population with low SES.

Our next step was to invite the parents of toddlers to a focus group interview to share and discuss the information that they were lacking and would find useful for improving the diet and food environment for their children. In total, five focus group interviews for parent groups were conducted, one at the university, one in a home setting, and three in settings where parents meet for other reasons (e.g., baby singing class). Two telephone interviews with mothers were conducted separately. Both parents attended the interviews conducted in the home. The remaining interviews were conducted among mothers only. Approximately 40% of focus group participants were non-native individuals. The most common questions and comments in interviews with users and health care nurses confirmed the main topics that were already planned for incorporation in the intervention. However, in line with the results of the interviews, the intervention was changed to include more focus than originally planned on spicy and exotic foods for the whole family, the right amount of different foods, self-eating skills, and family meal settings.

Based on a review of the literature, feedback from users, and discussions among the project group, the intervention was framed based upon three concepts: “the plate” (i.e., the food that is actually offered to the child), “the house” (referring to food that is available and accessible at home and parental feeding practices and food preparing skills), and “the grocery store” (parental awareness of the influence of environmental cues and how to make healthy choices).

### Website development

A prototype website was developed, and pilot tested with 14 participants in February 2017. The content of Food4toddlers was further refined based on this pilot test, recommendations from health authorities, and updated research in the field.

The website was developed using NEO Learning Management System. Two Masters students in Multimedia and Educational Technology at the University of Agder created a technical layout of the website, and the project group produced the content. The information provided on the website all relates to creating healthy food and eating environments for toddlers.

The homepage of the website contains an informational video about the website and information on why small changes in diet during the early years of a child’s life may be important in the long term. This page also gives some practical information on how to navigate the website and how to use the same information on a smartphone app. There is no difference in usability between the website and the smartphone app.

The website comprises four main elements: modules covering an introduction and seven topics on promoting healthy food and eating environments for the child, recipes, a discussion forum, and general information about food and beverages (the “Good to know” section), as shown in Table 2. When participants first accessed the website, not all content of the modules was visible to them, only the first two chapters. During the intervention period, access was expanded regularly (20 times) to include new content on the website; at this time, all the participants in the same wave received an e-mail with a link to the newly available information.

**Table 2:** Content of the intervention

Title	Explanation	Concept development
Modules	Topics are divided into modules with two to four subheadings (chapters). One general information module is also available.	1) Introduction to the intervention website with information about recipes, how to install the website app, and descriptions of the study. 2) The importance of early eating habits and how to interpret food labeling. A special focus on accessibility, availability, and variety of healthy food and beverages. 3) How taste develops and the importance of repeated exposures, basic tastes, and spicy food. 4) Self-feeding skills and children’s ability to self-regulate food intake. 5) Motivation to eat in a healthy way, being a good role model, and use of rewards. 6) Family meals: meal settings, preparing for meals, and meal composition. 7) Conscious and unconscious choices at home and in stores. 8) The benefits of children’s participation in cooking and encouragement to try new family dishes.
Recipes	A total 31 recipes are presented, 10 of which include an instructional video^a^	Dinner (17 recipes/5 videos),^a^ snacks (7/1), breads and cereals (5/3), and beverages (2/1).
Forum	The forum is divided into two sections: general questions and recipes.	Participants can ask questions and discuss relevant issues with each other. In the recipe forum, they can share recipes.
“Good to know”	Contains information about dietary issues relevant to the child’s age	Salt, honey, cinnamon, nitrites, potatoes, foreign foods (sushi), additives, and cod liver oil.

^a^One of the recipes with video was retrieved with permission from godfisk.no

### Modules

The first module contains information about the website and the project. The other seven modules contain two to four chapters. For each chapter, general information and tips and strategies to promote healthy behaviors are provided. In addition, one or two recipes, usually thematically linked to the topic, are recommended. The chapters also contains a video about unconscious choices while shopping [51], a game, eight quizzes, six explanatory figures, and some links to recommended websites (e.g., http://www.matportalen.no).

### Recipes

Out of a total of 31 recipes, 30 recipes were developed by three Masters students in Public Health at the University of Agder, in cooperation with the project group (Table 2). The focus for recipe development was to inspire the preparation of healthy meals for the whole family. The age span covered in the intervention is the period in which children should be able to eat the same foods as the rest of the family. The ingredients used should be available at a local supermarket. It was possible to print the recipes. For nine of the recipes, short instructional videos were developed to inspire parents to prepare the foods and to make the preparation process easier. The videos lasted from about 1 to 3 min and were produced by undergraduate students in Multimedia Technology and Design at the University of Agder.

### Discussion forum on the website

It was possible for participants to post questions and share information (e.g., recipes) with each other on a discussion forum. A project worker answered questions, usually within 3 working days. Participants who joined the same group had access to the same forum.

### “Good to know” information

In the interviews with health care nurses and end-users, some issues about special nutrients and dishes where discussed, including salt, nitrites, cinnamon, and foreign foods (such as sushi). We listed information about these issues together with information on honey, potatoes, food additives, and cod liver oil. The information given was based on National Health Authority recommendations.

### Behavioral change methods

Several behavioral change methods where included on the website, to improve the child’s diet through parental awareness of the child’s food and eating environments [52]. One method was belief selection. The messages on the website were designed to strengthen positive beliefs, weaken negative ones, and introduce new beliefs (i.e. reinforce the importance of family meals and highlight the importance of repetition of new foods) that are in line with the theory of planned behavior [53]. The active learning method included in this intervention are activity-based experiences; i.e. use of videos as a way to enhance cooking skills, as well as different quizzes [49]. Persuasive communication can include messages created in such a way as to be familiar and not too discrepant for participants [54]. The importance of small changes was highlighted on the website, and familiar settings were discussed (e.g., sitting as a family at the dining table). As mentioned, not all of the information on the website was immediately available to participants in the intervention group from the beginning. Revealing information gradually on the website over a span of time can enhance retention through repetition as well as the level of interest in and persuasiveness of the information [54]. Modeling is a method that can reinforce the desired action [49]. The website features videos with actors, who are in the same age group as participants, modeling desired behaviors. Our aim was to highlight barriers and facilitators and empower parents to make changes in their environment. The outcome might be that the environment for the child is created in a way that makes it easier to take action or reduces barriers to action [49].

### Measures and instruments

The primary outcome of this trial is the child’s overall diet and food variety (Table 3). Parents reported frequencies of intake of a variety of food normally eaten in Norway. Categorical scales ranging from 1) “never/less than every week” to 8) “five times a day” were used for food items from a national Food frequency questionnair (FFQ) [12] and 1) “never” to 6) “three times a day” for items from the MoBa study [55]. The secondary outcomes include parental feeding practices, family meal setting, food choice, awareness of the food environment, availability and accessibility of food in the home, food preparation and planning, and child weight and length. See Table 3 for specification about continuous and categorical variables. Most of the instruments used have previously been used in Norway or other countries and have been validated and in addition, some new questions were added. The new items about meal distraction are categorical variables with response alternatives from “disagree” to “agree” on a five-point scale: i.e. “I often look at the mobile phone during meals”. To measure the food environment three different categories of questions were used. The first type of questions relates to how available different foods are in the nearby shop and at home (i.e. fruit or whole grain biscuits) with response alternatives on a four-point scale from “not available” to “very available”. The second type of questions are statements on why they chose the way they do. The parents should respond on a 5 point-scale of “disagree” to “agree” on statements like: “I buy more if the shop is tidy and neatly organized”. The third category of questions include where in the house food is stored (“very accessible i.e. on the shelf”, “accessible i.e. in a cupboard/freezer/fridge”, “not accessible i.e. stored away in the basement/freezer/cupboard”). A test-retest was conducted in 2018 among 30 parents in kindergarten responding to these new questions twice with 2–3 weeks apart to test the reliability. The results showed a mean correlation of r = 0,551.

**Table 3:** Description of variables, measures, and instruments

Variable	Purpose of measure	Variable (Categorical/continuous)	Measure	Instrument	When to collect
PRIMARY OUTCOME					
Child’s diet	PSO, IC	Overall diet Food Variety (Continuous)	Food intake (core-and non-core foods) Healthy eating index	FFQ based on nationwide Norwegian diet survey among 12-month-old children [12] and the MoBa-study [55]	At baseline, 18, 24, and 48 months
SECONDARY OUTCOME Eating environment					
Child level: food preferences	SSO, IC	Food neophobia (Continuous)	Rating the child’s willingness to try new foods	The food neophobia scale [61]	At baseline, 18, 24, and 48 months
Parental level: feeding practices	SSO, IC	Feeding style and feeding practices (Categorical)	Under−/over-eating, hunger, infant cues. Feeding attitudes, practices, perceptions or concerns about weight	Comprehensive feeding practices [62, 63]	
	SSO, IC	Food neophobia (Continuous)	Rating the parent’s willingness to try new foods	The food neophobia scale [61]	
	SSO, IC	Self-efficacy (Categorical)	Parental self-efficacy in eating situations	Feeding self-efficacy [64]	
Family level: meal setting	SSO, IC	Frequency of shared meals (Categorical)	Frequency of meals and meal distractions	Questionnaires from the nationwide Norwegian diet survey among 12-month-old children [12] and items developed for this study	
Food environment					
Macro-level: grocery shopping	SSO, IC	Food choice and awareness of food environment (Categorical)	Planning, grocery shopping, what influences food choice	FCQ [65] SCQ: some elements made for this study, are based on theory of Wansink [51], and additional items developed for this study	At baseline, 18, 24, and 48 months
Micro-level: Home	SSO, IC	Availability and accessibility of food (Categorical)	Availability and accessibility of non-core and core foods Food preparation and planning	Questions developed for this study and items from Helland and colleagues [66]	
	SSO, IC	Meal management and food coping strategies (Categorical)	Self-efficacy related to meal management and food coping strategies	Meal management and food coping strategies questionnaire [67]	
OTHER					
Child anthropometrics	SSO	Anthropometric outcome (Continuous)	Height and weight	Self-reported, but measured at scheduled health center visits	At baseline, 18, 24, and 48 months
Parental characteristics	SC	Height and weight Demographics Socioeconomic status Food behaviors	Height and weight Education, occupation and food intake	Self-reported on questionnaire and simple FFQ [12]	
Website use	IC	Use of website by the intervention group	Usefulness and usability	Questions developed for this study, but include elements from Helle and colleagues [58]	At 18 months (intervention group)

*Abbreviations: PSO* primary study outcome, *SSO* secondary study outcome, *IC* intervention component, *SC* study covariate, *FFQ* food frequency questionnaire, *FCQ* food choice questionnaire, *SCQ* shopping choice questionnaire

### Other variables

The website contains information about which modules participants have used, how many times they have entered the modules, and the date and duration of each session. This information will form a part of the descriptive measurements in the study.

### Statistical analysis plan

For this protocol paper we present descriptive statistics of sample characteristics. All analyses were performed by using IBM SPSS Statistics 25.0.

Intervention effects on child diet, will be examined by use of mixed models (Linear Mixed Models and Generalized linear mixed models) with time as within factor (differences between baseline and post-test, follow-up 1, 2 and 3, respectively) and condition (intervention group, control group) as between-group factor. All models of pre-post outcomes will be adjusted for baseline values to account for regression to the mean effects. Using mixed models allows for use of incomplete data at the different follow-ups and thereby increase statistical power. We will present both crude and adjusted results. Intervention effects on both primary and secondary outcomes will be adjusted for the following variables: parental SES, BMI and age, and child gender and age (variables known from previous research to potentially confound such associations). To examine potential moderating effects such as parental SES (lower versus higher education level), a three-way interaction effect (time*condition*moderator) will be investigated for each outcome. As loss to follow up is expected, loss to follow-up-analyses will be performed, analyzing those lost to follow up compared to those remaining in the study. This will be done to identify if there are characteristics specific of those lost to follow up important to interpret the results.

The data will be stored securely on a password-protected computer with no connection between the data and personally identifiable information. The data will be available after project completion.

## Discussion

With increasing interest in and use of eHealth programs in health promotion, it is a high public health priority to determine what works best and in what context. For health promotion programs to be successful, it is suggested that interventions should be based on theory, include end-users and stakeholders, and have a randomized controlled design to establish effect.

This study protocol of the Food4toddlers intervention describes a randomized controlled trial targeting an important time span when the child’s preferences for food and eating habits are being established. Our project is in line with The Global Action Plan for the Prevention and Control of NCDs, focusing on early childhood intervention, and alerting and empowering parents in their role as gatekeepers of their child’s diet [56]. We expect that the intervention will provide parents with practical tools and make them more conscious of their child’s food and eating environments.

Laws and colleagues [57] compared three recruitment strategies in recruiting pregnant women or mothers with infants for an mHealth intervention; they found Facebook to be the best strategy. This is in line with the findings of a Norwegian eHealth study recruiting parents with children aged 3–5 months [58]. Facebook was the only recruitment tool used in Food4toddlers which might have a potential to recruit more low SES parents than other recruitment ways [57]. It was not very easy to recruit for this study as evidenced by a lower number of participants than initially aimed for. Because most parents with toddlers in Norway have already returned to work after having completed their maternity (or paternity) leave, we assumed that it would be more difficult to recruit for this study than for studies of younger aged children [58]. The time period from the participants signed up for recruitment (child age 7–12 months) until the baseline questionnaire was sent out, was up to five months, since the age of the child had to be 12 months at baseline. This might be one reason why there was a loss of participants from recruitment to baseline assessment.

Even though both fathers and mothers were invited to participate, only four fathers completed the baseline questionnaire. This is in line with respondents in other family-based interventions [59, 60]. The mother remains the main influence on the child’s diet [10] and can more easily engage in traditional non-technological interventions. Nevertheless, we had hoped that fathers would be engaged in this project as it uses an eHealth approach. The anthropometry measures were self-reported in this study. Measures by i.e. research staff would have increased validity of these data, but that was not possible in this study due to participants in all counties of Norway.

The findings of this study will enhance the understanding of how parents of toddlers access, use, collaborate with others, and engage in an eHealth intervention.

The benefits of participating in the intervention group include being updated on current information regarding healthy food and eating environments, and the possibility of improving their child’s diet quality and subsequent health. There are no foreseen risks related to participation in this study.

## Abbreviations

app: Mobile application
NCDs: Noncommunicable diseases
SES: Socioeconomic status
